# Information transfer in signaling pathways: A study using coupled simulated and experimental data

**DOI:** 10.1186/1471-2105-9-139

**Published:** 2008-03-04

**Authors:** Jürgen Pahle, Anne K Green, C Jane Dixon, Ursula Kummer

**Affiliations:** 1 Bioinformatics and Computational Biochemistry, EML Research, Schloss-Wolfsbrunnenweg 33, 69118 Heidelberg, Germany; 2 Department of Biological Sciences, The University of Warwick, Coventry, CV4 7AL, UK; 3 Leicester School of Pharmacy, De Montfort University, Leicester, LE1 7BH, UK; 4 Institute of Zoology/Bioquant, University of Heidelberg, Im Neuenheimer Feld 267, 69120 Heidelberg, Germany

## Abstract

**Background:**

The topology of signaling cascades has been studied in quite some detail. However, how information is processed exactly is still relatively unknown. Since quite diverse information has to be transported by one and the same signaling cascade (e.g. in case of different agonists), it is clear that the underlying mechanism is more complex than a simple binary switch which relies on the mere presence or absence of a particular species. Therefore, finding means to analyze the information transferred will help in deciphering how information is processed exactly in the cell. Using the information-theoretic measure transfer entropy, we studied the properties of information transfer in an example case, namely calcium signaling under different cellular conditions. Transfer entropy is an asymmetric and dynamic measure of the dependence of two (nonlinear) stochastic processes. We used calcium signaling since it is a well-studied example of complex cellular signaling. It has been suggested that specific information is encoded in the amplitude, frequency and waveform of the oscillatory Ca^2+^-signal.

**Results:**

We set up a computational framework to study information transfer, e.g. for calcium signaling at different levels of activation and different particle numbers in the system. We stochastically coupled simulated and experimentally measured calcium signals to simulated target proteins and used kernel density methods to estimate the transfer entropy from these bivariate time series. We found that, most of the time, the transfer entropy increases with increasing particle numbers. In systems with only few particles, faithful information transfer is hampered by random fluctuations. The transfer entropy also seems to be slightly correlated to the complexity (spiking, bursting or irregular oscillations) of the signal. Finally, we discuss a number of peculiarities of our approach in detail.

**Conclusion:**

This study presents the first application of transfer entropy to biochemical signaling pathways. We could quantify the information transferred from simulated/experimentally measured calcium signals to a target enzyme under different cellular conditions. Our approach, comprising stochastic coupling and using the information-theoretic measure transfer entropy, could also be a valuable tool for the analysis of other signaling pathways.

## Background

The simulation of complex biochemical networks has become very important to gain insight into the dynamic behavior of cellular processes [1-3]. Signaling pathways, in particular, often evade intuitive, and therefore rather static, explanations because of their highly nonlinear dynamics and many cross-links. However, despite the emergence of sophisticated high-throughput and *in vivo *imaging techniques, there is still a lack of high-quality single-cell multivariate data.

Such data would be very helpful in elucidating the nuts and bolts of many signaling mechanisms. In this study we use calcium signaling as an example. Calcium signaling represents one of the most versatile second-messenger pathways and, in many cell types, Ca^2+ ^(calcium) ions control a variety of cell functions from fertilization, secretion, enzyme activation and gene expression to cell death [4,5].

An intriguing fact is that, even in non-excitable cells like hepatocytes, the concentration of cytosolic calcium can display regular (spiking) or more complex (bursting) oscillations or prolonged elevated levels [6] after stimulation by an agonist and depending on the nature of this agonist. This oscillatory behavior is not only believed to save the cell from the toxic effects of sustained high cytosolic calcium levels and from desensitization, but has also been shown to increase the efficiency of calcium signaling [7]. In addition to these temporal patterns of calcium dynamics, interesting spatio-temporal patterns (e.g. calcium puffs and waves) have been described [4,8]. However, we will concern ourselves in this study with temporal patterns only.

Due to its importance for the functioning of many cell types and its interesting dynamics [9], calcium signal transduction has attracted numerous theoretical studies. Many different models of calcium signaling have been proposed, ranging from simple one-pool models [10] to more elaborate ones [11] incorporating many different processes. For a review on calcium models, see Schuster et al. 2002 [12].

Since a range of different agonists such as hormones (e.g. vasopressin) or nucleotides (e.g. ATP) trigger calcium responses and, on the other hand, a range of different targets (e.g. Ca^2+ ^dependent proteins such as calmodulin, CaM kinase II, protein kinase C, phosphorylase kinase or transcription factors e.g. NF-AT or NF-*κ*B) exist in the cell [13], specific information is likely to be encoded in the calcium signal and decoded again later on. It has been proposed that information might be encoded in the amplitude, frequency, duration, waveform or timing of calcium oscillations and the search for this *calcium code *has attracted a number of experimental and theoretical studies (for a review, see [14]).

On the experimental side, mainly the frequency decoding of spiking calcium oscillations has been examined. De Koninck and Schulman 1998 [15] demonstrated the sensitivity of immobilized CaM kinase II to Ca^2+^ oscillation frequency by *in vitro *rapid superfusion. Li et al. 1998 [16] found that NF-AT is activated optimally at a Ca^2+ ^oscillation frequency of about 1/min and Dolmetsch et al. 1998 [7] studied the differential regulation of T-cell NF-AT and NF-*κ*B by Ca^2+^oscillations of different frequencies. The interesting work of Oancea and Meyer 1998 [17] describes the activation of protein kinase C *γ *(PKC *γ*) by diacylglycerol (DAG) combined with high-frequency Ca^2+ ^spikes, which points to a joint code of calcium and DAG in that case.

Most theoretical studies also limit themselves to the spiking mode of calcium oscillations. Dupont et al. 2003 [18] could successfully reproduce the findings of [15] in a model. Gall et al. 2000 [19] examined the activation of liver glycogen phosphorylase by modeling a de-/phosphorylation cycle. Salazar et al. 2004 [20] studied the activation of target proteins by Ca^2+ ^oscillations in terms of efficiency, speed and specificity. Marhl et al. [21,22] investigated the decoding of time-limited calcium oscillations by downstream proteins. Recently, the bursting mode of Ca^2+^oscillations has been investigated by Larsen et al. 2004 [11] and Schuster et al. 2005 [23]. Using a simple model of calcium oscillations [11] and artificially generated calcium bursts [23] respectively to drive protein activation, these studies showed that specific information can be encoded in the waveform of bursting oscillations and thus that different proteins can be activated differentially at the same time. Rozi and Jia [24] studied the activation of glycogen phosphorylase by spiking as well as bursting calcium oscillations.

Even though information-theoretic measures [25] are in widespread use for physiological data [26] and neural information transfer [27], their application to biochemical systems is restricted to only relatively few studies. For instance, Prank et al. 1998 [28] and Kropp et al. 2005 [29] studied the encoding of hormonal signals in intracellular calcium signals using the so-called coding fraction and mutual information. The authors drive a deterministic model of calcium spiking with a specific form of generated noise and estimate the amount of information transferred. In [30] the same group couples a deterministic model of CaM kinase activation to experimentally measured data from HIT (hamster insulin-secreting tumor)-cells, but here the results are not analyzed in an information-theoretic manner.

We propose to use the information-theoretic measure transfer entropy [31] to estimate the information transferred by spiking or bursting calcium oscillations under different conditions. Transfer entropy has advantages over conventional measures such as (time-lagged) correlations, in that it detects all statistical dependencies (linear and non-linear), it is asymmetric, i.e. it distinguishes between information source and target, and it considers shared information due to a common history of the source and target by using conditioned transition probabilities. Transfer entropy has been applied to physiological data [26,32], financial time series [33], geological data [34] and others [35,36], but, so far, not to biochemical data. We used both simulated and experimentally measured time series for the estimation of transfer entropy. The simulated data were generated by a stochastic version of the simple calcium oscillations model in [37], extended by a stochastically simulated activation of target protein. We set up a framework for stochastic simulation of the calcium system, stochastic coupling of the enzyme activation process and estimation of the transfer entropy using kernel density estimation methods. We used this framework to investigate calcium information transfer in systems with different levels of activation and particle numbers.

Since multivariate experimental data is scarce, we devised a method, inspired by hybrid deterministic/stochastic simulation techniques, which allows the stochastic coupling of the enzyme activation process to arbitrary univariate calcium time series. We took experimental data from single-cell measurements on rat hepatocytes and coupled the activation of the stochastically simulated enzyme to them in order to get bivariate data. Finally we used these semi-experimental data as input for the estimation of the information transfer.

## Results

**Figure 1:**
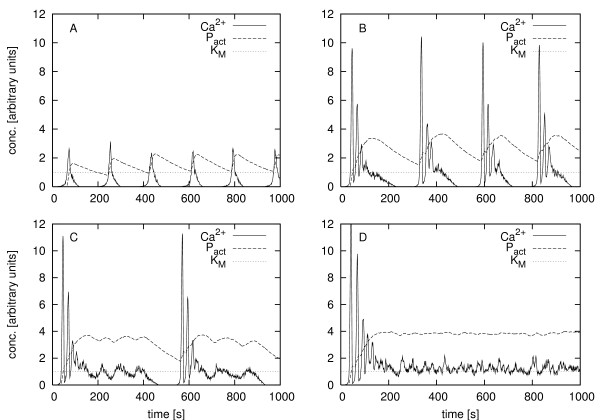
**Different calcium dynamics and coupled enzyme**. Coupling of the stochastically simulated activation of an enzyme to simulated calcium time series with different dynamical behavior according to Eq. (1). From top left to bottom right we see a spiking (A), bursting (B), irregular (C) behavior and overstimulation (D). *k*_2 _values 2, 2.85, 2.99 and 3.2, respectively, and volume 10^-12 ^[arbitrary units]. x-axis: time [s]. y-axis: concentration of Ca^2+ ^and the active form of the enzyme P_act_ and the enzyme's *K*_*M *_value.

In this study we used a simple receptor-operated model [37] with three variables (G_*α*_, PLC, cytosolic Ca^2+^) to generate calcium time series. This model was simulated stochastically by Gillespie's algorithm [38] (cf. Methods for details on the model and the stochastic simulation algorithm). Fig. 1 shows simulated time series of the Ca^2+^concentration under different cellular activation levels. The model is able to display understimulation (data not shown), spiking (panel A), bursting (panel B) and irregular behavior (panel C) as well as overstimulation (panel D). Spiking and bursting behavior is observed experimentally when hepatocytes are stimulated with vasopressin and ATP respectively.

The concentration of the active form of a simulated Ca^2+^-dependent enzyme, which was stochastically coupled to the calcium data, is also shown. We implemented a stochastic coupling scheme to be able to couple the simulated enzyme to arbitrary, simulated or experimental, calcium time series. This method is described in detail in Methods.

**Figure 2:**
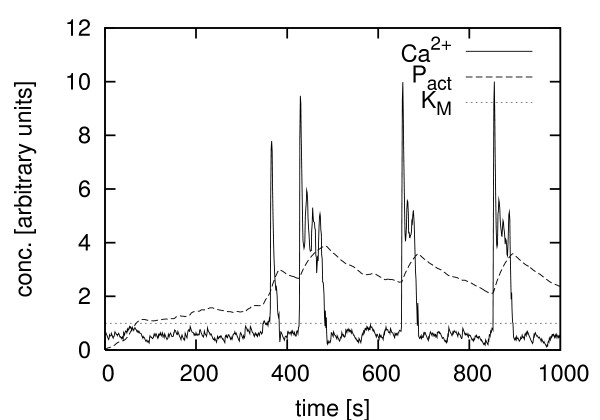
**Experimental data and simulated enzyme activation**. Coupling of the stochastically simulated activation of an enzyme to an experimentally measured calcium time series according to Eq. (1). Here the hepatocyte was stimulated using ATP (1.5 *μ*M). x-axis: time [s]. y-axis: concentration of Ca^2+ ^and the active form of the enzyme P_act _and the simulated enzyme's *K*_*M *_value (reaction volume of the simulated enzyme 10^-10 ^[arbitrary units]).

The coupling of the simulated enzyme to experimental data leads to semi-experimental time-series, one of which is shown in Fig. 2. Here an experimentally measured time series of the Ca^2+ ^concentration in a single rat hepatocyte (see Methods for further details) was computationally coupled to a simulated target enzyme according to Eq. (1). The hepatocyte was stimulated with ATP, which led to a bursting mode of calcium oscillations. The integrating character of the enzyme, which was shown elsewhere [11] to permit frequency decoding of the calcium oscillations, can easily be seen.

**Figure 3:**
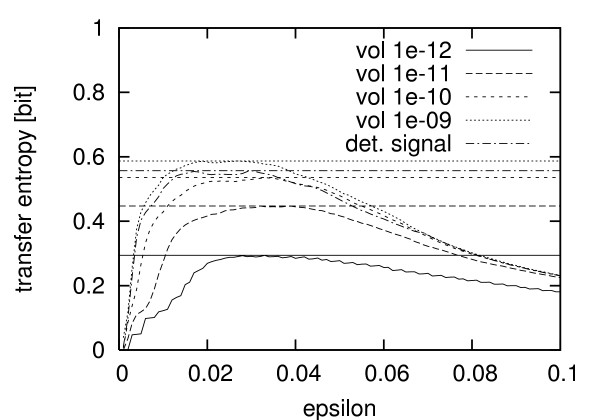
**Kernel density estimation of transfer entropy**. Scan of the estimated transfer entropies from Ca^2+ ^to active protein P_act _in the stochastically simulated system (*k*_2 _= 2.85, bursting). x-axis: *ε *values. y-axis: estimates of the transfer entropy in simulated systems of volumes 10^-12 ^to 10^-9 ^[arbitrary units] respectively. Also, the estimating process was applied to a deterministically simulated calcium signal (det. signal). In this case, the reaction volume of the (stochastically) simulated enzyme was 10^-10^.

Using these simulated and semi-experimental time series we investigated the information transferred from the calcium signal to the enzyme by estimating the transfer entropy (see Methods). In Fig. 3 an example of a scan over a range of bandwidths *ε *for the kernel density estimation is shown. The calcium system has been simulated in the bursting mode (*k*_2 _= 2.85) and with different values for the volume leading to different particle numbers. We used time courses of length 10000 s, sampled every second, after a transient of 10000 s has been cut off. For the density estimation we used a rectangular kernel and set the length of the Theiler window to 20 and the minimal number of neighbors to 5. As shown in the diagram, the estimates are biased towards zero for *ε *→ 0. For small *ε *values more and more samples are missing enough neighbors within the kernel bandwidth and those "lonely samples" are excluded from the estimation. For *ε *→ ∞ the kernel eventually covers the whole attractor, which also results in a value of zero for the transfer entropy. In between, there is a plateau-like range, where the estimate is almost independent of the *ε *value and which is supposed to be the best estimate of the true information transfer. We plotted the corresponding maxima in the diagram (horizontal lines). In the following we will always use those maximal values of the *ε *scans as estimates of the transfer entropy (see Discussion).

We also tested our estimation process by using surrogate data (constrained realizations, [39]). We estimated the transfer entropy of time series in which the temporal order of the calcium signal was destroyed by shuffling the samples (data not shown). This removed all dependencies, while the marginal probability distributions were preserved. Indeed, here the estimated transfer entropy showed values near zero (~0.02 – 0.07).

**Figure 4:**
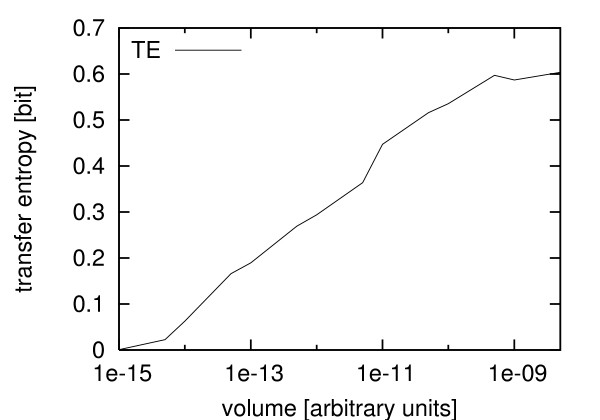
**Transfer entropy versus volume**. Maximum values of the estimated transfer entropy for different volumes in systems with *k*_2 _= 2.85 (bursting). x-axis: volume. y-axis: estimates of the transfer entropy.

To investigate how the information transfer changes with varying particle numbers in the system, we simulated the calcium model using a range of different volumes. Systems with low volumes, corresponding to low particle numbers, usually display strong random fluctuations, which could hamper the information transfer. Therefore our hypothesis was that a minimal number of particles are needed to allow for the faithful transfer of a certain amount of information. In fact, this is the case. Fig. 4 shows a scan of the transfer entropy of simulated systems in the bursting mode (*k*_2 _= 2.85) with different volumes. Here the information transfer increases with increasing volume (and particle numbers) until it seems to flatten out at about 0.6 bit/sample for volumes greater than 5 × 10^-10 ^[arbitrary units]. Interestingly, this corresponds to the particle numbers where the simulations display quasi-deterministic behavior [40]. With even higher volumes the system should eventually converge to the deterministic limit. In this case, also the coupling would be quasi-deterministic and the estimation of the transfer entropy should diverge (see Discussion). Therefore, regimes where the transfer entropy does not increase uniformly with increasing volume deserve further study, since this would be a helpful indicator that the transition to quasi-deterministic behavior is not uniform [40]. However, the huge computational cost prevented us from testing whether or not the apparent flattening is statistically significant in this case.

**Figure 5:**
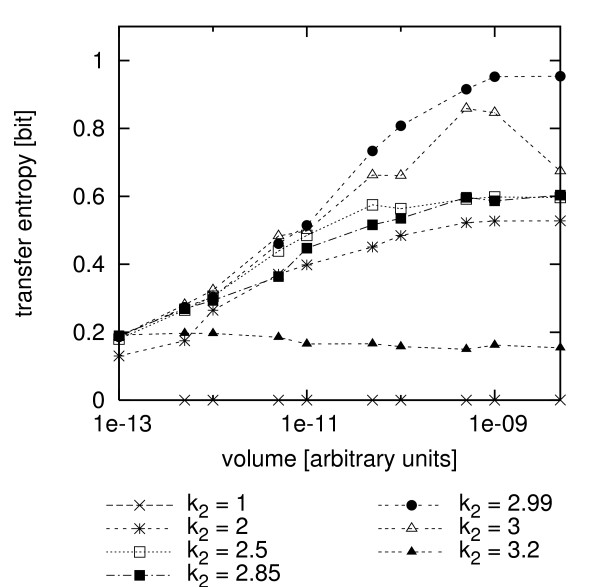
**Transfer entropy of different dynamic modes and volumes**. Maximum values of the estimated transfer entropy for different volumes and different *k*_2 _values corresponding to different dynamic modes (1 understimulation, 2 spiking, 2.5, 2.85 bursting, 2.99 irregular/elevated oscillations, 3, 3.2 overstimulation) in the simulated system. x-axis: volume. y-axis: estimates of the transfer entropy.

We also investigated the information transfer when the calcium system is in different dynamical modes (cf. Fig. 1). Fig. 5 shows a scan of transfer entropy estimates for different volumes (between 1 × 10^-13 ^and 5 × 10^-9^) where we varied the value of the bifurcation parameter *k*_2 _to get different dynamics, such as understimulation (*k*_2 _= 1), spiking (*k*_2 _= 2), bursting (*k*_2 _= 2.5, 2.85), irregular behavior/elevated oscillations (*k*_2 _= 2.99) and overstimulation (*k*_2 _= 3, 3.2).

In the case of under- or overstimulation (*k*_2 _= 1 or *k*_2 _= 3.2), the system is in a (noisy) steady state and this results in low values for the transfer entropy. For *k*_2 _= 1 the calcium concentration is near its resting level, which is far below the *K*_*M *_value of the enzyme. No enzyme gets activated and no information can be transferred. For *k*_2 _= 3.2 the calcium steady state lies above the enzyme's *K*_*M *_value and the amount of active enzyme reaches its maximum. In contrast to understimulation, here the information transfer is not exactly zero, even though it takes low values of ~0.2. The reason for this is that now the noisy steady state is near the *K*_*M *_value of the enzyme and it can pick up some random fluctuations in calcium concentration. If the system is in an oscillatory mode, such as spiking (*k*_2 _= 2) or bursting (*k*_2 _= 2.5, 2.85), the transfer entropy increases with increasing volume until it seems to flatten out for volumes above 5 × 10^-10^, as shown above.

An interesting effect can be observed for *k*_2 _= 2.99 and *k*_2 _= 3, where the deterministic limits of the calcium dynamics are elevated oscillations and an elevated steady state, respectively. However, the stochastic system shows irregular behavior with small volumes. For high volumes, oscillations are observed even for *k*_2 _= 3. For both parameter values, the generally very high level of transfer entropy is due to the position of the center of their oscillations. It is near the *K*_*M *_value of the enzyme, so that the enzyme is responsive even to minute variations in the calcium level. Interestingly, for *k*_2 _= 3 the transfer entropy shows a maximum at the volumes 1 × 10^-9 ^and 5 × 10^-10^. An explanation for this effect is that for the higher volume 5 × 10^-9^, the system is already near the deterministic limit, which is just a rather uninteresting elevated steady state with relatively low information transfer. On the other hand, for smaller volumes, the information transfer gets degraded because of increasing stochastic fluctuations. Those fluctuations are especially pronounced in this parameter range, because the sensitivity of the system (measured by the divergence) is high (see [40] for details). Those two opposed trends lead to a maximum in a range where the system is still oscillatory, but not yet too noisy.

**Figure 6:**
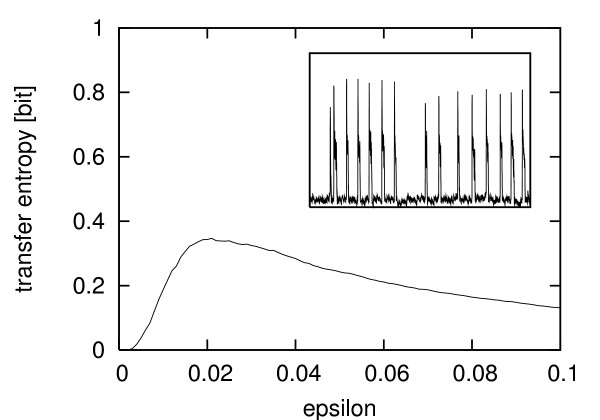
**Transfer entropy of semi-experimental data**. Scan of the estimated transfer entropy from an experimentally measured Ca^2+ ^time series (3904 s) to the simulated target enzyme P_act _according to Eq. (1). Here the hepatocyte was stimulated using ATP (1.5 *μ*M). x-axis: *ε *values. y-axis: estimates of the transfer entropy. (reaction volume of the simulated enzyme 10^-10 ^[arbitrary units]).

If we look at the estimates for a volume of 5 × 10^-9 ^only (the biggest systems considered in this study), there is a slight increase in estimated transfer entropy from spiking to increasingly complex bursting oscillations (see Table 1). The transfer entropy is very high for elevated oscillations near the enzyme's *K*_*M *_value and it drops to a very low value in the case of an elevated steady state, e.g. overstimulation. Intuitively, one would think that the information transfer should be correlated to the complexity (spiking, bursting or irregular oscillations) of the calcium oscillations, since more complex input signals can potentially carry more information. However, this can only be hinted at from our experiments. One should be wary not to over-interpret the absolute numbers, since we found them very much dependent on the estimation process used. Also, they are subject to statistical fluctuations. Furthermore, the enzyme is most sensitive for calcium levels near its *K*_*M *_value. For the input signal to generate a high information transfer, it is important to meet that range. The transfer entropy nicely detects this for the oscillatory regime with *k*_2 _= 2.99 and high volumes, where the oscillations exactly meet the *K*_*M *_value. Here the estimated transfer entropy is high, even though the dynamics is apparently less complex than in the bursting mode. To compare simulations with experimental data we coupled an experimentally measured calcium time series from a single hepatocyte to the stochastic model of enzyme activation. In this case the cell was stimulated using 1.5 *μ*M ATP and showed bursting behavior (see Fig. 6, inset). We monitored the calcium concentration over a time period of 3904 s (one sample per second). The reaction volume of the simulated enzyme was set to 10^-10 ^[arbitrary units]. For the kernel density estimation, we used a Theiler window of length 20 and reduced the minimal number of neighbors to 2 because of the smaller number of samples available. Fig. 6 shows a scan of the transfer entropy estimates from this semi-experimental time series over a range of *ε *values. The estimated transfer entropy has a maximum at about 0.35 bit.

**Table 1:** **Different dynamical behavior and maximal transfer entropy values.** Maximum values of the transfer entropy (TE) for different stimulation strengths *k*_2 _and their respective dynamic regime in the simulated system with volume 5 × 10^-9^.

*k*_2_	Dynamic behavior	TE
1	Understimulation	0.00
2	Spiking	0.52
2.5	Bursting	0.59
2.85	Bursting	0.60
2.99	Elevated oscillations	0.95
3.2	Overstimulation	0.15

**Figure 7:**
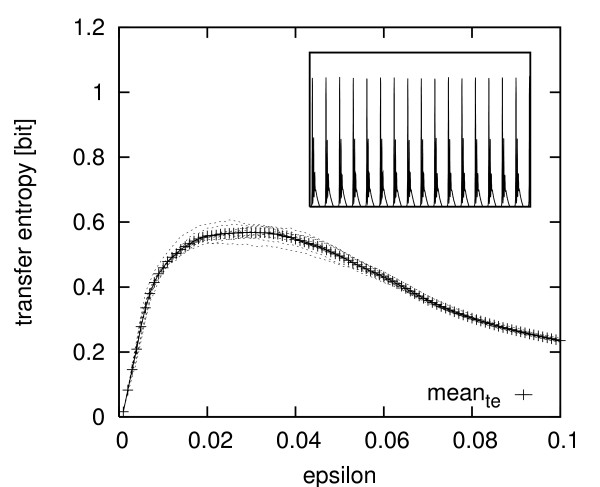
**Transfer entropy scan**. Scan of the estimated transfer entropies from Ca^2+^ to active enzyme P_act _in the stochastically simulated system with *k*_2 _= 2.85 and 10^-10 ^volume. Shown are the estimates for ten different time series of length 3904 s each and their mean value. x-axis: *ε *values. y-axis: estimates of the transfer entropy.

For a direct comparison, we calculated 10 stochastically simulated calcium time series of length 3904 s showing bursting behavior (*k*_2 _= 2.85). One of them can be seen in the inset of Fig. 7. We then coupled these time series to the same enzyme process and estimated the transfer entropy using the same set of parameters as before. We plotted the results of the 10 different simulations plus the mean value in Fig. 7. The mean of the estimated transfer entropies has a maximum of about 0.57 bit. The variance of the estimated values is biggest in the plateau region with a maximum in standard deviation of approximately 0.03.

The significantly higher transfer entropy values of the simulated system can partly be explained by the existence of two episodes in the experimental data without bursts (The calcium-mobilizing agonist was absent from the experimental medium for the duration of these two episodes). We removed these episodes and repeated the estimation which yielded a transfer entropy maximum of roughly 0.39 bit. An explanation for the remaining discrepancy is that the simulated bursts have a considerably longer duration than the bursts in real hepatocytes. Therefore, the calcium signal spends more time within the sensitive region of the enzyme (near the *K*_*M *_value) which clearly increases information transfer.

## Discussion

In the following we will motivate the choice of several technical elements as well as discuss their strengths and limitations.

### Stochastic coupling procedure

Stochastic fluctuations in cellular systems are not just random noise, but can even change the dynamics of the system [41] as was seen, for instance, in our simulations for parameter values near bifurcation points (*k*_2 _= 2.99 and small volumes). Therefore it is important to consider random effects (and the effects of the system size on those fluctuations) when modeling systems with relatively low particle numbers, e.g. signal transduction pathways.

It should be noted here that, even in those cases where stochastic effects do not change the dynamics significantly, deterministic coupling of a biochemical reaction system to experimental data [30] is not appropriate for our purposes. The estimation of transfer entropy diverges for coarse-grained continuous systems and increasing resolution if the coupling between the processes is deterministic [31]. Therefore our stochastic coupling scheme of the simulated enzyme to calcium time series is absolutely essential for this study.

Since the experimentally measured calcium concentration is only known at a discrete set of points in time and therefore we assumed it to be constant between two samplings, the coupling of a simulated enzyme to those time series can only be an approximation. However, it is apparent that, in the limit of a sampling frequency of the given time series near the frequency of reaction events in the system and a measurement resolution in the range of single particles, our method converges to the mathematically exact solution. For nearly every practical case, neither the number of samples nor the resolution will satisfy these theoretical conditions. To make sure that this fact did not compromise our results, we compared simulated data where the enzyme was only coupled to a calcium time series with data that was calculated by exact stochastic simulation of the whole system, i.e. calcium dynamics plus enzyme activation, and where no approximation was involved (data not shown). For the parameter values and sampling times we used, our results were not changed considerably by the approximate coupling.

One shortcoming of the stochastic coupling procedure described here is that it is a one-way process. Obviously, the input calcium time series is fixed and can not be changed during the process and so possible feedback of the target enzyme on the calcium system, e.g. calcium buffering by proteins or feedback via protein kinase C, has to be neglected.

### Choice of model parameters

The volume of a hepatocyte is about 2 pL [42]. Assuming that the cytosol, where the free Ca^2+ ^is located, takes up about half of the total volume of the cell and that, in the case of bursting, the calcium level peaks around 1 *μ*M, this results in a particle number of about 600 000. This particle number roughly corresponds to a volume of 10^-10 ^in the arbitrary units of the calcium model used. Therefore our results lie well in the range of physiologically meaningful parameters. Also the parameters of the simulated enzyme have been chosen to be, at least, biologically plausible. Most of the time calcium binding to enzymes occurs cooperatively, as e.g. with calmodulin. Calmodulin has four binding sites with high affinity (*K*_*d *_≈ 0.1 – 1 *μ*M) for Ca^2+^. For this reason, we, like the authors of other numerical studies [11,23,30], employ a Hill term of 4th order. The *K*_*M *_value of the simulated enzyme lies between the calcium resting level and the amplitude of secondary peaks, in the case of bursting oscillations.

The reason for choosing this calcium model instead of a more detailed one was that, even though it is very simple in terms of size and kinetic functions, it can show both spiking and bursting behavior in addition to (elevated) steady states, thereby mimicking the dynamics of real cells after stimulation by different agonists (see [37] for details). Most other models cannot show bursting oscillations. It also was relatively easy to implement and fast to simulate stochastically. Nevertheless, the generation of some of the time series with high particle numbers required computation times in the range of several days. In fact, the purpose of this study was not to analyze this specific calcium model and therefore the approach presented here is not restricted to that model. It should also be mentioned that our framework can easily be applied to arbitrary enzyme regulation mechanisms, provided that they allow stochastic simulation of the Gillespie type, i.e. a propensity can be assigned to every possible event in the system.

One problem of the calcium model we used is that the amplitudes of the oscillations vary for different dynamic modes (see Fig. 1), whereas in real hepatocytes the amplitudes of calcium oscillations have been reported to be independent of the type of oscillations. Also the duration of bursts is longer than in experiments which, we believe, led to the discrepancy in transfer entropy between simulated and experimental data. To mitigate these issues we plan to use more realistic calcium models with more constant oscillation amplitudes, e.g. [11] in the future.

### Estimation of transfer entropy

Often, (time-lagged) correlations are used to quantify the coherence of two observables. However, correlations can only indicate linear relations, not non-linear ones. Therefore mutual information has been developed which is sensitive to all statistical dependencies [43]. Unfortunately, this measure is still (like correlations) symmetric and cannot distinguish between information sources and targets.

The transfer entropy, on the other hand, is explicitly asymmetric because it uses conditioned transition probabilities. As stated by Schreiber [31, p. 461], "*transfer entropy *is able to distinguish effectively driving and responding elements and to detect asymmetry in the interaction of subsystems." In addition, the use of transition probabilities makes it a dynamic measure, meaning that it can account for the history of the processes. This, together with its ability to consider linear and non-linear dependencies, renders it appropriate for use on non-linear signal transduction systems.

We found that a major issue with this measure is that it is not trivial to estimate it from time series in a reliable way and that the estimation is quite data-intensive.

One crucial point is that the processes have to be ergodic to allow for the estimation of the probability densities from one time series alone. Also they must be Markovian. In other words, their histories of length *k *and *l *(see Methods), which are taken into account, must be longer than possible correlation times. This is very important, because the transfer entropy detects the deviation from the Markov property. One simple example where this condition would not be fulfilled is when we just reversed the direction and estimated the transfer entropy from the enzyme signal to the calcium signal (P_act _→ Ca^2+^). We saw already that in our setting there can be no feedback from the enzyme to the calcium system and thus no information can be transferred this way. Because the transfer entropy is a directional measure and can distinguish between information transferred in one and the other direction, one would naively think that it should equal zero (plus statistical fluctuations) here. This, however, is not the case, because the calcium signal alone is not Markovian. In fact, in the model it is coupled to G_*α *_and PLC and their influence will lead to a transfer entropy which is not zero. There are two possible solutions to this issue: a) consider the whole system (Ca^2+^, G_*α *_and PLC) or condition on all coupled subsystems, or b) take into account a long enough history for the processes in which all relevant information is already embedded.

In all practical applications of the transfer entropy, especially with purely experimental data, one has to fix the lengths of the two signal histories (*k *and *l*) with care. Since the characteristic time-scale of auto-dependencies in measured data is not known a priori, they can not be regarded as stemming from an order-one Markov process. Therefore, one should estimate the transfer entropy using different values for the order of the underlying processes, and longer histories should be preferred. However, the often very limited amount of data renders this avenue infeasible in many cases, since kernel estimation would have to be applied to distribution functions in four and more dimensions. One possible resort here would be coarse-graining of the time series and the use of the discrete version of the transfer entropy. In the present study we restricted ourselves to the order-one case, the reason being that, in our case, the coupled protein is actually described by a Markov process of order one and is not dependent on previous values. Therefore, a history length of 1 (*k *= *l *= 1) suffices.

Kernel density estimation is known to be very dependent on the choice of a correct kernel bandwidth *ε*. Rules of thumb exist for the optimal bandwidth of (univariate) Gaussian kernels [44] which, however, are said to often lead to oversmoothing. Little has been done for multivariate kernels however. Instead of just using one bandwidth, we scanned the estimated transfer entropy over a range of different *ε *values and checked for the range of bandwidths where the estimates are independent of *ε*, e.g. a plateau is visible in the scans (Fig. 3). If there is a definite plateau, its values are simultaneously the maximal values of the scan. Due to this and because the estimated transfer entropy was observed to underestimate the true value [45], we chose to take the maxima of the scans as estimates of the transfer entropy.

The calcium signal and the enzyme signal have different ranges of values. Therefore we normalized the time series to have mean 0.0 and standard deviation 1.0 prior to the estimation, which allowed us to use the same *ε *in both spaces. This is justified, because the (continuous) transfer entropy is independent of coordinate transformations [45].

To improve our calculations, we used a Theiler window approach and excluded all estimates where only less than a required minimal number of neighbors could be found. This avoided spurious effects caused by temporal correlations and dampened statistical fluctuations, respectively. In this study we mainly used rectangular kernels. However, we also tried Gaussian kernels (data not shown), which did not change our results considerably.

Transfer entropy is an averaged measure, i.e. it describes the information transfer over the whole observation interval. We observed that periods in the experimental calcium time series without bursts (Fig. 6) decreased the overall transfer entropy. Therefore, if the processes under study are expected to show some kind of locking or unlocking episodes, which we would dub *statistical locking*, the measure would have to be calculated on smaller (disjoint or overlapping) windows in order to see possible changes over time. Care has to be taken, though, that the windows are big enough to get a sound statistical basis for the estimation.

We want to stress that the absolute values of our transfer entropy estimates are, of course, dependent on the parameters of the estimation procedure. In particular, the minimum number of neighbors needed for a sample to be considered plays a major role here. Setting this number to values greater than 1 helps to diminish statistical fluctuations, but can create a bias towards zero if there are not enough samples available. Therefore, one should be cautious when interpreting these values and should not mix results coming from different estimation procedures without justification. We only compared estimates where the estimation parameters, the type of kernel and the length of the input time series were the same. We attributed the discrepancy in estimated transfer entropy of simulated and experimentally measured data to lacking realism of the simple calcium oscillations model used. Hence, we note here that transfer entropy could very well be employed as a measure of realism for signaling pathway models. We envisage its use in biochemical modeling where models are optimized so as to have the same information transfer as observed in experiment.

Also, regarding the rates of information transfer we estimated in this study, one should be cautious. Even though they can provide a useful basis for hypotheses on the functioning of cellular signal transduction, it is not known what fraction of the information that can maximally be transferred is actually used by downstream cellular processes. Because it is not yet clear what features of the calcium signal really carry relevant information, we used an information-theoretic approach. It potentially measures all the information from the calcium signal that can be found in the protein signal. In addition, this model-free approach facilitates direct comparisons between simulated and experimentally measured data.

Nevertheless, specific information transferred from calcium to cellular processes could, in principle, be estimated by extending the simple model to include these processes under consideration and estimating the transfer entropy directly between calcium and the observables of these processes. This includes the detection, analysis and quantification of possible cross-talk between different signaling pathways.

### A general framework

It should be mentioned that there are many potential variants and extensions of the estimation algorithm (simple or adaptive histograms, adaptive kernel density estimation, likelihood estimators and others [44]), which we could not cover here. However, regardless of the algorithm used, the basic strength of the information-theoretic approach is that it is model-free. This allows the direct comparison of simulated and experimental data.

## Conclusion

In this study we combined methodologies from different fields in order to elucidate the cellular information transfer via Ca^2+ ^signaling. The main ingredients we used are:

• Modeling and simulation of calcium signal transduction, in particular stochastic approaches.

• Stochastic coupling of a Ca^2+^-dependent protein to experimental and simulated data.

• The so-called transfer entropy introduced by Schreiber [31] and its estimation using kernel density estimation techniques.

We developed and implemented a framework for the analysis of both simulated and experimentally measured Ca^2+ ^time series with the information-theoretic measure transfer entropy. This involved the stochastic coupling of a simulated enzyme to arbitrary calcium time series and the estimation of the transfer entropy of those bivariate data using kernel density estimation methods.

We investigated the information transfer from the calcium signal to the target enzyme under different conditions, namely different particle numbers (by varying the volume) and different calcium dynamics (corresponding to different stimuli). We found that, most of the time, information transfer increases with increasing particle numbers in the system. However, this increase is different in systems with different dynamic modes (spiking, bursting, etc.). More complex dynamic modes (bursting or irregular oscillations) tend to result in higher values of the transfer entropy. We observed that the input signal has to lie in the sensitive range, e.g. near the *K*_*M *_value of the enzyme, for the information transfer to be efficient. We also estimated the transfer entropy based on experimental data from hepatocytes. The values of these estimates are significantly lower than those from comparable simulated data. The major reason for this seems to be the unphysiologically long duration of simulated bursts. Further study is needed to investigate that in detail.

Even though the estimation of transfer entropy from time series is tricky and there are still some unsolved issues, it is a promising tool not only for the quantification of information transfer in biochemical networks, but also, for instance, to distinguish between different stochastic time series where a pure visual investigation is difficult. The direct comparison of two stochastic trajectories is difficult: Not the actual trajectory is important, but the features of it, that are essential for the correct functioning of the cell. In the case of calcium signaling, they are the ones that can be decoded by downstream elements.

Each dynamic state exhibits its own sensitivity to random fluctuations [40] and this should be reflected in the faster degradation of information transfer if the sensitivity is high. Therefore, one possible application of this approach could be the detection of the transition between stochastic and quasi-deterministic behavior, in cases where it is difficult to be identified by visual inspection alone. We saw one example of that already in the case of *k*_2 _= 2.99 (see Results), where the stochastic behavior is qualitatively different from the deterministic limit and where the transfer entropy could detect this transition. Another application could be information theory-based model fitting where models are optimized so as to have the same information transfer as observed experimentally.

It is worth mentioning that our framework is not at all limited to calcium signaling. Stochastic coupling and/or estimation of transfer entropy from biochemical data can be easily applied to other biochemical models and pathways.

Our approach can also be extended in a number of ways. On the technical side, for example, the estimation of transfer entropy from limited data sets should be improved. This could include the automatic determination of an optimal kernel bandwidth, the use of different kernels or alternative probability density estimation methods.

On the biological side, we plan to investigate the type and amount of information carried by the different properties of the calcium signal (amplitude, frequency, duration, shape, timing), because it is not yet clear which of those are really used in cells. For instance, enzymes which cannot decode a specific signal should lead to a transfer entropy value of almost zero. On the contrary, the transfer entropy is expected to show significant higher values for enzymes which are sensitive to the input signal. Thus we hope that the transfer entropy can give valuable hints for further theoretical and experimental studies. Furthermore, we want to use our framework to study different enzyme regulation mechanisms and to analyze other signaling pathways including their possible cross-talks.

## Methods

### Model

In this study we used a simple receptor-operated model [37] with three variables (G_*α*_, PLC, cytosolic Ca^2+^) to generate calcium time series (cf. Table 2 for a list of reactions and corresponding kinetic functions). This model was simulated stochastically by Gillespie's algorithm [38]. Because the original model has arbitrary units, we scaled it in time (by 1/20) to have roughly the same frequency as observed experimentally. This scaling corresponds to a division of the rate parameters *k*_1_, *k*_2_, *k*_3_, *k*_5_, *k*_7_, *k*_8_, *k*_10 _and *k*_11 _by 20.

**Table 2:** **Model of calcium oscillations.** Simple model of calcium oscillations [37]. Parameters: *k*_1 _= 0.212, *k*_3 _= 1.52, *K*_4 _= 0.19, *k*_5 _= 4.88 *K*_6 _= 1.18, *k*_7 _= 1.24, *k*_8 _= 32.24, *K*_9 _= 29.09, *k*_10 _= 13.58, *k*_11 _= 153, *K*_12 _= 0.16. *k*_2 _is bifurcation parameter and is set to different values in simulations depending on the desired behavior.

Reactions			Kinetics
	→	G_*α*_	*v*_1 _= *k*_1_
	$\overset{{\text{G}}_{\alpha }}{\to }$		*v*_2 _= *k*_2 _× [G_*α*_]
G_*α*_	$\overset{\text{PLC}}{\to }$		$\nu_{3}=\frac{k_{3}\times [{\text{G}}_{\alpha }]\times [\text{PLC}]}{K_{4}+[{\text{G}}_{\alpha }]}$
G_*α*_	$\overset{{\text{Ca}}^{\text{2+}}}{\to }$		$\nu_{4}=\frac{k_{5}\times [{\text{G}}_{\alpha }]\times [{\text{Ca}}^{2+}]}{K_{6}+[{\text{G}}_{\alpha }]}$
	$\overset{{\text{G}}_{\alpha }}{\to }$	PLC	*v*_5 _= *k*_7 _× [G_*α*_]
PLC	→		$\nu_{6}=\frac{k_{8}\times [\text{PLC}]}{K_{9}+[\text{PLC}]}$
	$\overset{{\text{G}}_{\alpha }}{\to }$	Ca^2+^	*v*_7 _= *k*_10 _× [G_*α*_]
Ca^2+^	→		$\nu_{8}=\frac{k_{11}\times [{\text{Ca}}^{\text{2+}}]}{K_{12}+[{\text{Ca}}^{\text{2+}}]}$

The parameter *k*_2 _represents the stimulation strength and serves as bifurcation parameter to vary the dynamic behavior of the model. In addition, we changed the numbers of particles present by varying the volume of the system.

Coupled to this simple signal generating model is a model for calcium binding to a protein. In the following we will use a slight modification (the amount of inactive protein is not assumed to be constant) of the regulation mechanism described in [11].

(1) $$\begin{array}{l} \frac{{\text{dP}}_{\text{act}}}{\text{d}t}=\frac{k_{\text{act}}\times {\text{Ca}}_{\text{cyt}}^{p}}{K_{M}^{p}+{\text{Ca}}_{\text{cyt}}^{p}}\times {\text{P}}_{\text{inact}}-k_{\text{inact}}\times {\text{P}}_{\text{act}}\\ {\text{P}}_{\text{tot}}={\text{P}}_{\text{inact}}+{\text{P}}_{\text{act}}=\text{constant} \end{array}$$

Activation of the inactive form of protein P_inact _to its active form P_act _by Ca^2+ ^is modeled by a Hill term of order four while deactivation follows mass action kinetics (Eq. (1)). The parameters were set to *k*_act _= 0.025, *k*_inact _= 0.005, *K*_*M *_= 1.0, P_tot _= 5.0 and *p *= 4.

### Stochastic simulation and coupling

Since the seminal work of Gillespie 1976 [38,46] several algorithms have been proposed to calculate trajectories governed by the Chemical Master Equation [47,48]. These trajectories are instances of the underlying stochastic process and consider the random fluctuations correctly. The different methods are mathematically equivalent and differ only in their algorithmic implementation. There are a number of software packages supporting stochastic simulation [49,50].

For each type of reaction *R*_*μ *_(1 ≤ *μ *≤ *M*, with *M *being the number of reactions) in the system, a propensity *а*_*μ *_is assigned, such that *а*_*μ*_*dt *describes the probability of the reaction to occur within the next infinitesimal time interval of length *dt*. *а*_*μ *_is a product of a specific stochastic reaction rate, which usually can be derived easily from the conventional reaction rate, and a combinatorial term, that depends on the stoichiometry of the reaction. Gillespie derives a reaction probability density function (Eq. (2)) as the basis for his so-called Direct Method.

(2) $$P(\tau ,\mu |x,t)=a_{\mu }(x)\exp\left(-\sum_{1..M}a_{\mu }(x)\tau \right)$$

This function describes the probability *P*(*τ*, *μ*|*x*, *t*) that starting in state *x *at time-point *t *the next reaction in the system will occur after time *τ *and will be of type *R*_*μ*_. One can see that the reaction times are exponentially distributed and thus the process is a homogeneous Poisson process. By iteratively drawing pseudo-random numbers according to this reaction probability density function and updating the system state, trajectories can be calculated in a Monte Carlo scheme.

Since we not only want to analyze simulated calcium dynamics, but also intend to couple measured calcium time series to our enzyme activation model, we have to take that influence into account. The coupled calcium system exerts an influence on the reaction propensities *а*_*μ *_in the protein model and thus they can no longer be considered constant between two reaction events. Mathematically, this is equivalent to changing the homogeneous Poisson process into an inhomogeneous one and therefore the pure stochastic simulation methods cannot be used in this case.

The reaction probability density function for such systems with time-dependent *а*_*μ *_reads (cf. [51]):

(3) $$P(\tau ,\mu )=a_{\mu }(t+\tau )\exp\left(-\int_{t}^{t+\tau }\sum_{1..M}a_{\mu }(t)dt\right).$$

One can sample this inhomogeneous Poisson process by integrating the differential reaction probabilities over time. Whenever a stopping criterion for one of the reactions is reached, the integration is interrupted and the corresponding reaction event is instantiated. This method has been used in hybrid stochastic/deterministic simulation methods [52,53], where the set of reactions is partitioned into a stochastically simulated and a deterministically simulated subset. During the simulation, the influence of the (fast) deterministic subset on the stochastic subset has to be considered.

When we couple a time series to a stochastically simulated system, we do not know the states of the system which produced the time series between two samples. Therefore a reasonable presumption is to assume piece-wise constant particle numbers between two sample times. In this special case we can use, for instance, Gillespie's Direct Method, with the modification that recalculation of all the *a*_*μ *_in the system, which are dependent on the coupled time series, is needed whenever a new sample is observed in the time series. This approximation is discussed in section Discussion.

We implemented this simple method in C++-code dynamically linked to Octave (Version 2.9.9 on Linux) [54]. Our implementation accepts time series with arbitrary sampling times, both evenly and unevenly sampled.

### Transfer Entropy

The so-called transfer entropy is an information-theoretic [25] measure proposed by Schreiber 2000 [31] to quantify the dependence of one stochastic process on a second one. Its definition for discrete systems *I *and *J *reads as follows:

(4) $$T_{J\to I}=\sum p(i_{n+1},i_{n}^{\left(k\right)},j_{n}^{\left(l\right)})\log\frac{p(i_{n+1}|i_{n}^{\left(k\right)},j_{n}^{\left(l\right)})}{p(i_{n+1}|i_{n}^{\left(k\right)})}.$$

The transfer entropy has Kullback-Leibler divergence form and measures the deviation of process *I *from its Markov process behavior of order *k *due to the interaction with process *J*. In this study, we set *k *= *l *= 1, which is justified in section Discussion. One should keep in mind though, that in the general case longer history lengths might be required. Setting those parameters correctly is crucial for a reliable estimation. If this is the case, probability densities in spaces of dimension >3 must be estimated. For the estimation of the transfer entropy, usually either the time series is coarse-grained by histogram-based methods and the transfer entropy is estimated on the symbolic time series or kernel density estimation [44] methods are used.

We implemented a kernel density estimation method for the transfer entropy [45] in C++-code, which has been dynamically linked to Octave (Version 2.9.9 on Linux) [54]. For the estimation of local probability densities, we mainly used a rectangular kernel with variable radius *ε *(Eq. (5)):

(5) $$\begin{array}{c} {\hat{p}}_{\{x_{i}\},\varepsilon }(x_{i})=\frac{1}{N\varepsilon }\sum_{n=1}^{N}K\left(\frac{x_{i}-x_{i}(n)}{\varepsilon }\right)\\ K(r)=\frac{1}{2}\Theta (1-|r|) \end{array}$$

with Θ the Heaviside function.

To avoid spurious effects caused by temporal correlations, we employed a Theiler window approach which excluded all neighbors that were too close in time. In addition, in order to dampen statistical fluctuations, only samples that had a user-defined minimal number of neighbors were considered. The kernel density estimation procedure was implemented using a two-dimensional box-assisted neighbor-searching algorithm [55], which resulted in a five to six fold speed-up compared to the naive implementation. We scanned the transfer entropy of the simulated calcium model and the coupled enzyme for different volumes (between 1 × 10^-13 ^and 5 × 10^-9 ^[arbitrary units]), corresponding to different particle numbers in the system (roughly between 600 and 30 000 000 during primary peaks), and for different dynamics, e.g. different values of the bifurcation parameter *k*_2 _(1 understimulation, 2 spiking, 2.5, 2.85 bursting, 2.99 irregular, 3, 3.2 overstimulation). The same kernel bandwidth were used in the calcium and the protein concentration spaces, but the data was normalized to have mean 0.0 and standard deviation 1.0 prior to the estimation.

### Experiments

Single hepatocytes were isolated from fed male Wistar-strain rats (150–250 g) by collagenase perfusion as described previously [56]. The cells were harvested and incubated at 37°C at low density (10^3 ^cells/ml) in 2% type IX agarose in William's medium E (WME). Single hepatocytes were prepared for microinjection with the bioluminescent Ca^2+ ^indicator aequorin as described previously [57]. The injected cell was transferred to a perfusable cup held at 37°C, positioned under a cooled, low-noise photomultiplier, and continuously superfused with WME, to which agonists were added. Photon counts were sampled every 50 ms by computer. At the end of an experiment, the total aequorin content of each cell was determined by discharging the aequorin by lysing the cell. The data were normalized retrospectively by computer, by calculating the photon counts per second divided by the total counts remaining. The computed fractional rate of aequorin consumption could then be plotted as [Ca^2+^]_*i *_using in vitro calibration data and exponential smoothing with time constants: for resting [Ca^2+]^_*i*_, 12 s; for transients, 1 s.

In addition, we transformed these data to be roughly in the same range of values ([0, 10]) as that of the simulated data.

### Materials

Aequorin was provided by Prof. O. Shimomura (Marine Biological Laboratory, Woods Hole, MA, U.S.A). Collagenase was obtained from Roche Diagnostics (Lewes, U.K.) and WME from Invitrogen (Paisley, U.K.). Agarose and agonists were purchased from Sigma-Aldrich (Poole, U.K.).

## Authors' contributions

JP conceived of the study, carried out all calculations and drafted the manuscript. AKG and CJD performed the experimental measurements. UK participated in conception of the study, interpretation of the results and drafting the manuscript. All authors read and approved the final manuscript.

## References

[B1] Endy D, Brent R (2001). Modelling cellular behavior. Nature.

[B2] Érdi P, Tóth J (1989). Mathematical models of chemical reactions – Theory and applications of deterministic and stochastic models.

[B3] Heinrich R, Schuster S (1996). The Regulation of Cellular Systems.

[B4] Berridge M, Bootman M, Lipp P (1998). Calcium – a life and death signal. Nature.

[B5] Carafoli E (2002). Calcium signaling: A tale for all seasons. PNAS.

[B6] Woods N, Cuthbertson K, Cobbold P (1986). Repetitive transient rises in cytoplasmic free calcium in hormone-stimulated hepatocytes. Nature.

[B7] Dolmetsch R, Xu K, Lewis R (1998). Calcium oscillations increase the efficiency and specificity of gene expression. Nature.

[B8] Falcke M (2003). Deterministic and stochastic models of intracellular Ca^2+ ^waves. New Journal of Physics.

[B9] Keener J, Sneyd J Mathematical Physiology.

[B10] Somogyi R, Stucki J (1991). Hormone-induced Calcium Oscillations in Liver Cells Can Be Explained by a Simple One Pool Model. J Biol Chem.

[B11] Larsen A, Olsen L, Kummer U (2004). On the encoding and decoding of calcium signals in hepatocytes. Biophys Chem.

[B12] Schuster S, Marhl M, Höfer T (2002). Modelling of simple and complex calcium oscillations – From single-cell responses to intercellular signalling. Eur J Biochem.

[B13] Celio M, (Ed) (1996). Guidebook to the Calcium-Binding Proteins.

[B14] Larsen A, Kummer U, Falcke M, Malchow D (2003). Information Processing in Calcium Signal Transduction. Understanding Calcium Dynamics.

[B15] De Koninck P, Schulman H (1998). Sensitivity of CaM Kinase II to the Frequency of Ca^2+ ^Oscillations. Science.

[B16] Li WH, Llopis J, Whitney M, Zlokarnik G, Tsien R (1998). Cell-permeant caged InsP_3 _ester shows that Ca^2+ ^spike frequency can optimize gene expression. Nature.

[B17] Oancea E, Meyer T (1998). Protein Kinase C as a Molecular Machine for Decoding Calcium and Diacylglycerol Signals. Cell.

[B18] Dupont G, Houart G, Koninck PD (2003). Sensitivity of CaM kinase II to the frequency of Ca^2+ ^oscillations: a simple model. Cell Calcium.

[B19] Gall D, Baus E, Dupont G (2000). Activation of the Liver Glycogen Phosphorylase by Ca^2+ ^Oscillations: a Theoretical Study. J Theor Biol.

[B20] Salazar C, Politi A, Höfer T, Mamitsuka H, Smith T, Holzhütter H, Kanehisa M, DeLisi C, Heinrich R, Miyano S, Kyoto (2004). Decoding of calcium oscillations by phosphorylation cycles. Proceedings of Fourth International Workshop on Bioinformatics and Systems Biology.

[B21] Marhl M, Perc M, Schuster S (2005). Selective regulation of cellular processes via protein cascades acting as band-pass filters for time-limited oscillations. FEBS Letters.

[B22] Marhl M, Perc M, Schuster S (2006). A minimal model for decoding of time-limited Ca^2+ ^oscillations. Biophys Chem.

[B23] Schuster S, Knoke B, Marhl M (2005). Differential regulation of proteins by bursting calcium oscillations – a theoretical study. BioSystems.

[B24] Rozi A, Jia Y (2003). A theoretical study of effects of cytosolic Ca^2+ ^oscillations on activation of glycogen phosphorylase. Biophys Chem.

[B25] Weaver W, Shannon C (1949). The Mathematical Theory of Communication.

[B26] Imas O, Ropella K, Ward B, Wood J, Hudetz A (2005). Volatile anesthetics disrupt frontal-posterior recurrent information transfer at gamma frequencies in rat. Neuroscience Lett.

[B27] Borst A, Theunissen F (1999). Information theory and neural coding. Nature Neuroscience.

[B28] Prank K, Schöfl C, Läer L, Wagner M, von zur Mühlen A, Brabant G, Gabbiani F (1998). Coding of time-varying hormonal signals in intracellular calcium spike trains. Pac Symp Biocomput.

[B29] Kropp M, Gabbiani F, Prank K (2005). Differential coding of humoral stimuli by timing and amplitude of intracellular calcium spike trains. IEE Proc-Syst Biol.

[B30] Prank K, Läer L, von zur Mühlen A, Brabant G, Schöfl C (1998). Decoding of intracellular calcium spike trains. Europhys Lett.

[B31] Schreiber T (2000). Measuring information transfer. Phys Rev Lett.

[B32] Katura T, Tanaka N, Obata A, Sato H, Maki A (2006). Quantitative evaluation of interrelations between spontaneous low-frequency oscillations in cerebral hemodynamics and systemic cardiovascular dynamics. Neuroimage.

[B33] Marschinski R, Kantz H (2002). Analysing the information flow between financial time series – An improved estimator for transfer entropy. Europ Phys J B.

[B34] Materassi M, Wernik A, Yordanova E (2007). Determining the verse of magnetic turbulent cascades in the Earth's magnetospheric cusp via transfer entropy analysis: preliminary results. Nonlin Processes Geophys.

[B35] Nichols J, Seaver M, Trickey S, Salvino L, Pecora D (2006). Detecting impact damage in experimental composite structures: an information-theoretic approach. Smart Mater Struct.

[B36] Lungarella M, Sporns O (2006). Mapping Information Flow in Sensorimotor Networks. PLOS Comp Biol.

[B37] Kummer U, Olsen L, Dixon C, Green A, Bornberg-Bauer E, Baier G (2000). Switching from Simple to Complex Oscillations in Calcium Signaling. Biophys J.

[B38] Gillespie D (1976). A General Method for Numerically Simulating the Stochastic Time Evolution of Coupled Chemical Reactions. J Comp Phys.

[B39] Schreiber T, Schmitz A (2000). Surrogate time series. Physica D.

[B40] Kummer U, Krajnc B, Pahle J, Green A, Dixon C, Marhl M (2005). Transition from Stochastic to Deterministic Behavior in Calcium Oscillations. Biophys J.

[B41] Rao C, Wolf D, Arkin A (2002). Control, exploitation and tolerance of intracellular noise. Nature.

[B42] Savage V, Allen A, Brown J, Gillooly J, Herman A, Woodruff W, West G (2007). Scaling of number, size, and metabolic rate of cells with body size in mammals. PNAS.

[B43] Steuer R, Kurths J, Daub C, Weise J, Selbig J (2002). The mutual information: Detecting and evaluating dependencies between variables. Bioinformatics.

[B44] Silverman B (1986). Density estimation for statistics and data analysis.

[B45] Kaiser A, Schreiber T (2002). Information transfer in continuous processes. Physica D.

[B46] Gillespie D (1977). Exact Stochastic Simulation of Coupled Chemical Reactions. J Phys Chem.

[B47] Gibson M, Bruck J (2000). Efficient Exact Stochastic Simulation of Chemical Systems with Many Species and Many Channels. J Phys Chem A.

[B48] Cao Y, Li H, Petzold L (2004). Efficient formulation of the stochastic simulation algorithm for chemically reacting systems. J Chem Phys.

[B49] Copasi. http://www.copasi.org.

[B50] Dizzy. http://magnet.systemsbiology.net/software/Dizzy.

[B51] Gillespie D (1992). Marcov Processes – An Introduction for Physical Scientists.

[B52] Salis H, Kaznessis Y (2005). Accurate hybrid stochastic simulation of a system of coupled chemical or biochemical reactions. J Chem Phys.

[B53] Alfonsi A, Cancès E, Turinici G, Di Ventura B, Huisinga W (2005). Adaptive Simulation of Hybrid Stochastic and Deterministic Models for Biochemical Systems. ESAIM Proceedings.

[B54] Octave. http://www.octave.org.

[B55] Kantz H, Schreiber T (1997). Nonlinear Time Series Analysis.

[B56] Dixon C, Cobbold P, Green A (1995). Actions of ADP, but not ATP, on cytosolic free Ca^2+ ^in single rat hepatocytes mimicked by 2-methylthioATP. Br J Pharmacol.

[B57] Cobbold P, Lee J, McCormack J, Cobbold P (1991). Aequorin measurements of cytoplasmic free calcium. Cellular Calcium: A Practical Approach.

